# Hydrodynamic Trails Produced by *Daphnia*: Size and Energetics

**DOI:** 10.1371/journal.pone.0092383

**Published:** 2014-03-26

**Authors:** Lalith N. Wickramarathna, Christian Noss, Andreas Lorke

**Affiliations:** Institute for Environmental Sciences, University of Koblenz-Landau, Koblenz-Landau, Germany; University of Hull, United Kingdom

## Abstract

This study focuses on quantifying hydrodynamic trails produced by freely swimming zooplankton. We combined volumetric tracking of swimming trajectories with planar observations of the flow field induced by *Daphnia* of different size and swimming in different patterns. Spatial extension of the planar flow field along the trajectories was used to interrogate the dimensions (length and volume) and energetics (dissipation rate of kinetic energy and total dissipated power) of the trails. Our findings demonstrate that neither swimming pattern nor size of the organisms affect the trail width or the dissipation rate. However, we found that the trail volume increases with increasing organism size and swimming velocity, more precisely the trail volume is proportional to the third power of Reynolds number. This increase furthermore results in significantly enhanced total dissipated power at higher Reynolds number. The biggest trail volume observed corresponds to about 500 times the body volume of the largest daphnids. Trail-averaged viscous dissipation rate of the swimming daphnids vary in the range of 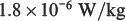 to 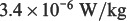 and the observed magnitudes of total dissipated power between 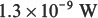 and 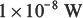, respectively. Among other zooplankton species, daphnids display the highest total dissipated power in their trails. These findings are discussed in the context of fluid mixing and transport by organisms swimming at intermediate Reynolds numbers.

## Introduction

Small-scale fluid motion and mixing induced by swimming zooplankton in aquatic ecosystems have important physiological and ecological consequences at organism and population scale. The flow field around the organisms affects feeding strategies and feeding success [1], [2] as well as the reception and dispersal of chemical [3], [4] and hydro-mechanical cues [5], which allow for detecting prey or predators [6]. Fluid transport and mixing by swimming zooplankton also has been considered as a potentially significant energy source for vertical mixing in density-stratified waters on global scales [7]–[9]. The extent to which zooplankton-generated flow can contribute to vertical mixing, however, was argued to be insignificant due to the small spatial scales at which currents are produced [10], [11].

Theoretical analysis [12] and numerical simulations [13] on copepods suggest that the highly fluctuating flow field around their beating feeding appendages and swimming legs is damped by viscosity and high-frequency temporal fluctuations are restricted to spatial scales, which are smaller than the viscous length scale 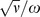 (with 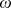 and 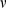 being the angular frequency of the beating appendages and the kinematic viscosity respectively). Beyond this length scale, a steady flow field develops, which depends on organism Reynolds number (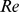). If 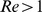, i.e. if inertia of the displaced fluid surpasses viscous forces, an increasing fraction of total power is dissipated at spatial scales exceeding the size of the organism. In fact, 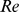 can be considered as a relative trail length because it scales with the ratio of length scale over which hydrodynamic disturbances dissipate to organism size [14]. However, energy dissipation provides only one possible measure of the size of the footprint of swimming zooplankton. Because the molecular diffusivities of dissolved substances are much smaller than the diffusivity of momentum, which is described by the kinematic viscosity, the corresponding concentration fluctuations are more persistent and are dissipated at much larger spatial scales [15].

Existing laboratory and numerical studies on the size and the structure of hydrodynamic footprints of swimming zooplankton have mainly focused on copepods [6], [16], [17]. The different feeding strategy and resulting swimming patterns of other highly abundant zooplankton species of similar size, such as *Daphnia*, can, however, be expected to result in different hydrodynamic footprints [1].

Locomotion of the filter-feeding daphnids is attained by a pair of extended appendages (second antennae) and erected swimming hairs during the downward directed power stroke that generates more drag than folded appendage and collapsed swimming hairs during the upward recovery stroke [18]. Beating of the second antennae with typical frequencies of 3–5 Hz [19], [20] produces the thrust required to propel the 0.2–5 mm sized organism forward. This length scale is about 0.2 mm for a beating antenna of a *Daphnia*.

The typical organism (body) Reynolds number of *Daphnia* is about *O* 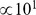 to 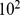 [18], [21] and similar to those of copepods [18], [22], [23]. The size of the hydrodynamic trails of swimming *Daphnia* has been investigated in laboratory experiments by Gries et al. [20] as a function of density stratification. They observed that the volume of the wakes is much larger than the organism itself. The optical Schlieren technique applied in their measurements, however, required very large density gradients, which were demonstrated to strongly affect trail length. In weak density stratification Noss and Lorke [24] have recently observed enhanced dissipation rates of kinetic energy in the trail of a freely swimming daphnid on spatial scales exceeding the size of the organism by two orders of magnitude.

In this study we analyze a series of laboratory measurements of the trajectories and hydrodynamic footprints produced by freely swimming *Daphnia* of different sizes in the absence of density stratification and background flow. By quantifying the spatial dimensions of enhanced kinetic energy dissipation rates in the trail of the swimming organism, we provide experimental evidence for the ubiquitous existence and 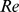 dependence of highly-energetic flow structures exceeding the size of the organism swimming at intermediate 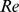. The intermediate 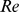 in our study refers to 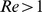 (viscous flow), but not fully turbulent (i. e. not 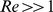).

## Materials and Methods

### Organisms and Measurements

All test organisms of species *Daphnia magna* were cultured following standard regulatory requirements [25]. For the measurements, groups of 5–20 organisms of the same age (5, 20, and 25 days old, respectively) were inserted into the test aquarium and allowed to sufficiently adapt to the test environment. For each age group, the core body length 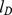 (head to the proximal end of the caudal spine [26]) was estimated for 5–10 different organisms from selected images. The average growth rate estimated for the culture was 0.072 mm/day, and can be considered as typical for *D. magna* [26].

The test aquarium with a cross-sectional area of 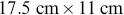 and a height of 16 cm was submerged in a larger temperature-controlled aquarium to prevent the generation of convective currents due to slight fluctuations of room temperature. The aquarium was illuminated from above with a dimmable natural white LED-panel, while light intensity was adjusted (568 lm) so that it provides sufficient illumination for organism tracking. Swimming behavior of organisms was not affected by the white light because, given the size of the aquarium in our system, white light was homogenously distributed with negligible attenuation. As previously reported [27]–[29], the swimming behavior depends on the rate of change in light intensity but not on the magnitude of light intensity itself.

**Figure 1:**
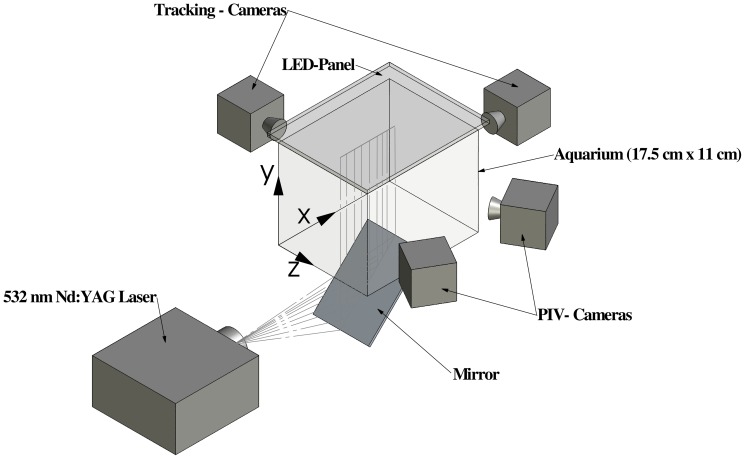
A three-dimensional depiction of the experimental set-up. Temperature fluctuations potentially causing convective currents in the test aquarium were suppressed by placing it into an outer aquarium of constant temperature, which is not shown here.

We deployed two cameras for tracking in combination with two stereoscopic PIV (Particle Image Velocimetry) cameras for three-dimensional velocity measurements. Swimming trajectories of all daphnids were tracked using two orthogonally arranged CCD-cameras (FlowSense4M, Dantec Dynamics, four-megapixel, 8 bit greyscale resolution) mounted on bi-telecentric lenses (TC 4M, Opto Eng.) having a focal depth of 5.6 cm (Figure 1). The usage of bi-telecentric lenses provided a pixel resolution of about 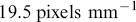, which is independent of location within the sampling volume. The spatial resolution of each camera-lens combination was measured using a custom-made calibration target.

Three-dimensional current velocities were measured within a vertical plane located in the center of the test aquarium using stereoscopic PIV. The plane was illuminated by short laser pulses (Litron Nano L 200-15 PIV double pulse laser, wavelength: 532 nm, pulse duration: 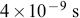), and the displacement of seeding particles (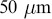 diameter Polyamide particles, Dantec Dynamics) was observed from two different perspectives (Figure 1). The laser light sheet had a thickness of 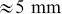, and it should be noted that the swimming behavior was not affected by the green laser light (Table S1 provided in the supporting information). A four-megapixel greyscale CCD-camera (FlowSense4M, Dantec Dynamics) and a two-megapixel greyscale PCO camera (HiSense 610, Dantec Dynamics) were used for the PIV measurements. Video S1 (provided in the supplementary information) exemplifies a sequence of raw images showing particle displacements by freely swimming daphnids of different sizes.

The timing of laser pulses and image acquisition of all four cameras were controlled using *Dantec Dynamicstudio* software (version 3.20). The two stereoscopic PIV cameras captured images during the exposure with the laser light sheet, while two tracking cameras captured images during the time window in which the laser light sheet was off. Images from all four cameras were recorded at 14.8 Hz for 5.6 min.

## Data analysis

### Swimming trajectories and patterns

Three-dimensional swimming trajectories of all daphnids within the test aquarium were estimated following the procedure described by Noss et al. [30]. The raw tracks were initially refined to a minimum length to screen out a large number of very short segmented tracks, and furthermore, tracks which did not cross the laser light sheet were discarded. Moreover, near-wall segments of the refined trajectories were also discarded because daphnids tend to veer from the primary swimming trajectory in the neighborhood of the glass walls. Consequently, the lengths of swimming trajectories chosen for further analysis were typically about 60 mm.

Instantaneous swimming speeds of the organisms were estimated using the distances between subsequent positions along the swimming trajectory. Mean swimming speeds 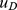 were obtained from averaging of instantaneous speeds over the entire trajectory. Body Reynolds number of daphnids 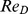 was calculated using mean swimming speed and body length (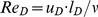, where 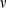 is the kinematic viscosity of water at 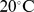).

**Figure 2:**
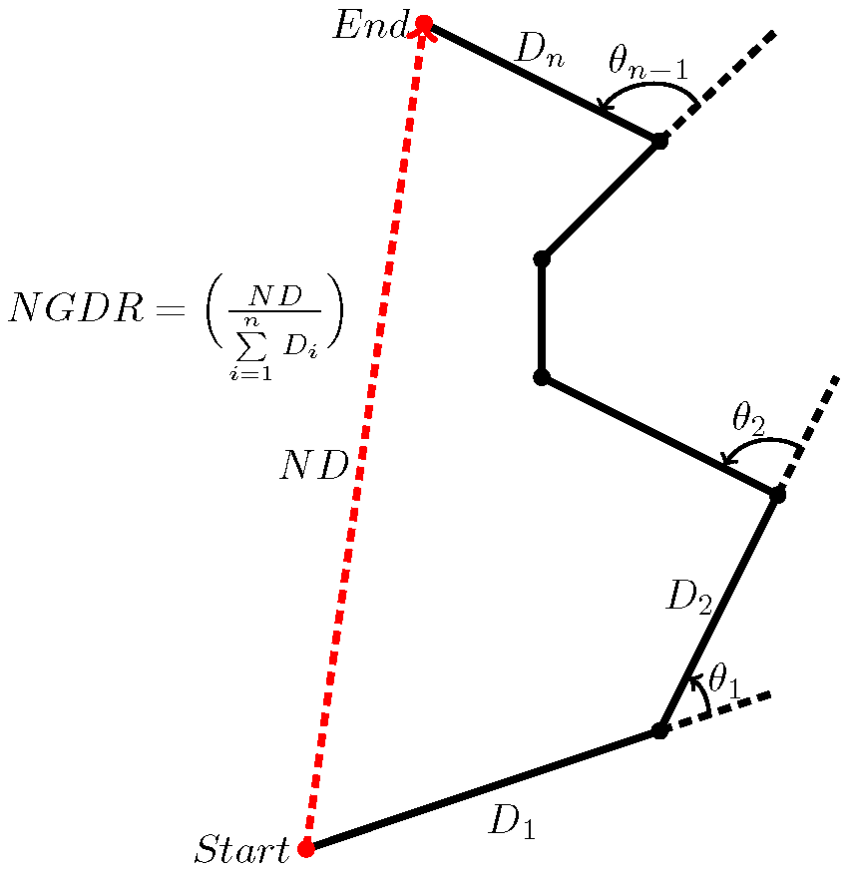
An illustration of different metrics in characterizing swimming trajectories. The mean displacement is the mean of distances 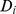 traveled between two subsequent observations (black dots). The net displacement 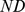 is the straight-line distance between the initial and final locations, and the gross displacement is the sum of distances 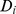. The mean turning angle is the trigonometric mean of angles 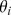 formed by changes in direction between observations [32].

**Figure 3:**
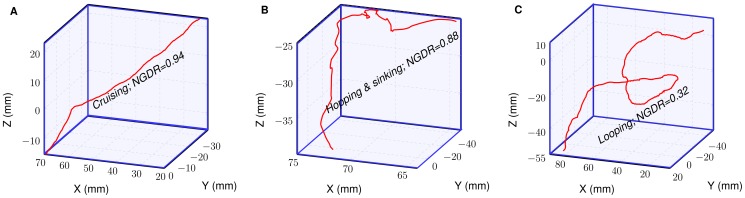
An illustration of swimming patterns of *Daphnia*. The swimming patterns can be distinguished based on NGDR values specified.

Observed swimming trajectories were further used to categorically characterize the swimming pattern of organisms. A variety of metrics have been proposed for differentiating swimming patterns [31] (Figure 2). All proposed measures (e.g. path length or turning angle) are scale-dependent and no single measure may characterize swimming paths unambiguously [32]. Since both aspects of path length and turning angle are embodied, the Net to Gross Displacement Ratio (NGDR) within a distance of 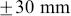 from the light sheet was adopted in the present study. Three typical swimming patterns were discriminated based on observed NGDR values (Figure 3):

- Cruising (NGDR: 1.0–0.9): In this swimming pattern, organisms swim in a near-straight line trajectory (Figure 3A).
- Hopping and sinking (NGDR: 0.9–0.6): In this pattern of swimming, organisms tend to discretely ascend and descend from their pathways (Figure 3B).
- Looping (NGDR: 0.6–0.25): In this particular swimming pattern, organisms distinctly display a swirling or spiral-like motion (Figure 3C).

### Analysis of trail

Three-dimensional current velocity vectors within the laser light sheet were obtained from stereoscopic PIV analysis [33] by using an adaptive correlation method [34] available in DynamicStudio software (version 3.20, Dantec Dynamics). The spatial and temporal resolution of the final velocity estimates are 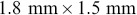 and 0.0676 s respectively.

Dissipation rate of turbulent kinetic energy [35] was estimated from measured velocity components using;
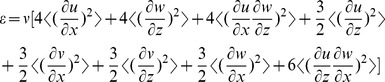
(1)


Where 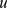, 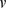, and 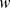 denote the current velocity components in 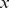, 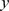, and 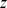 directions, respectively (Figure 1) and velocity gradients were estimated using a central difference scheme [36].

**Figure 4:**
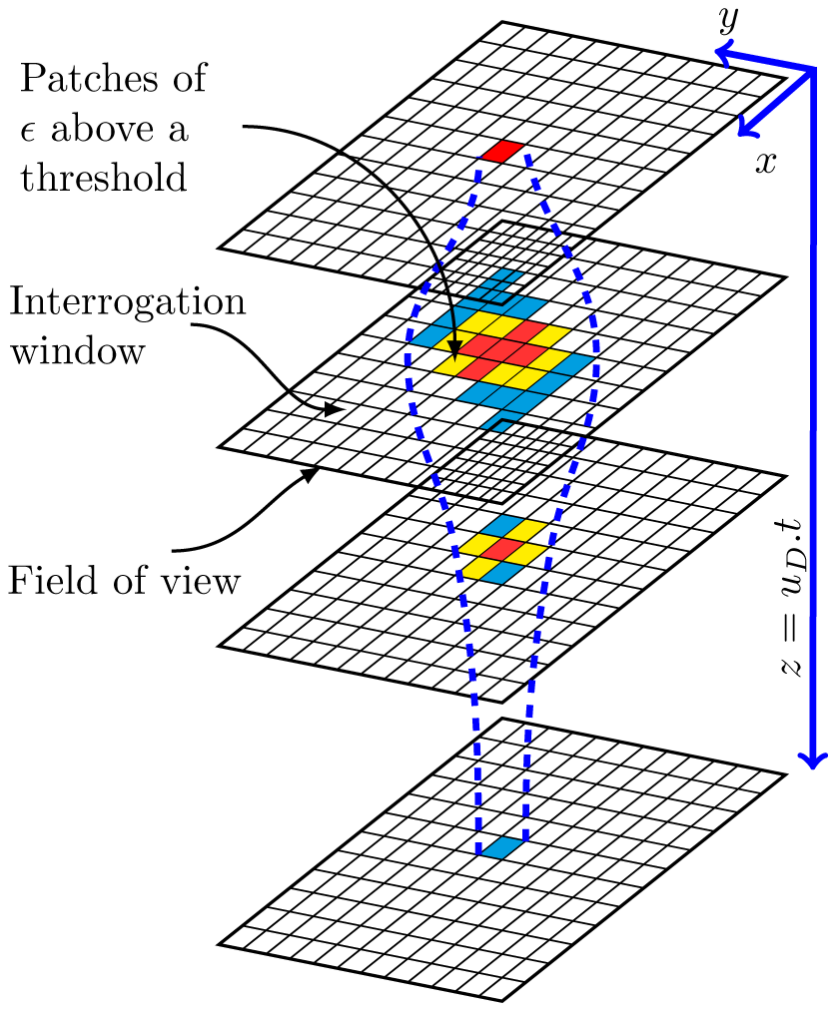
A 3D schematic diagram that illustrates the method of estimating the volume of dissipation rates. The gridded windows indicate the field of view at different time stamps across the laser light sheet while smaller grids represents spatial window of the interrogation area (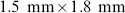). A *Daphnia* swims in the 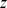-direction, and three different grid colors indicate different levels of dissipation rates induced by the swimming *Daphnia*. The 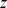-coordinate was determined as the product of *Daphnia* swimming velocity (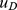) and time taken to passed through field of view (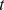) while the trail cross-sectional area was computed by the total area of patches of 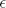 above our threshold for each PIV image. The three-dimensional distribution of energy dissipation rates in the trails was reconstructed by the product of 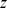-coordinate and the trail cross-sectional areas. Blue dashed lines represent the boundary of the trail.

The cross-sectional area of fluid disturbances induced by swimming daphnids crossing the laser light sheet were identified using a threshold of 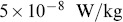 in energy dissipation rates [24]. It should be noted that the threshold was somewhat arbitrarily chosen depending on the resolution and noise in our measurements. Trail cross-sectional area was estimated as the total area featuring dissipation rates above this threshold for individual PIV images. The three-dimensional distribution of energy dissipation rates in the trails was reconstructed by estimating the unresolved *z*-coordinate as the product of *Daphnia* swimming velocity obtained from tracking and the time elapsed after it has passed through the PIV field of view (Figure 4). The trail volume was estimated by integrating the measured planar dissipation distributions along over all three spatial dimensions. Figure S1 (provided in the supporting information) shows an example of a trail produced by a cruising *Daphnia*. Assuming a cylindrical trail shape, equivalent trail diameters (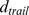) were estimated using the observed trail lengths (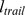) and trail volumes (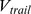). Mean dissipation rates of kinetic energy 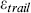 and total dissipated power 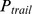 were obtained from the log-average of observed dissipation rates within each trail [37] and from the multiplication of the log-averaged dissipation rates with trail volume times water density 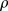, respectively.

For reference, we calculated the wake length of a sphere (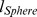) sinking in a still surrounding based on velocity scaling in a laminar wake region [38], [39]. Using the velocity distribution provided in [40] (their Equation 5), 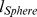 was estimated (Equation 2) as the distance from a sphere having the diameter 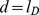 and moving at speed 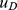 at which the centerline velocity (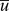 in Equation 2) has decreased to 10% of its maximum value. Thus, the wake length can be formulated as;
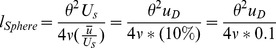
(2)


Where 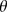 initial momentum thickness of a wake (Equation 1 of [40]), 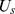 is the sphere velocity (*Daphnia*) and 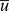 the mean streamline velocity at distance 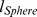.

Total dissipated power of the swimming daphnids was further estimated using an approach of Huntley and Zhou [9] for a global assessment of kinetic energy dissipation by swimming organisms. By applying their approach to our observations, the rate of energy utilization 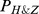 (in W) to overcome drag acting on a daphnid can be calculated as:
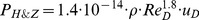
(3)


Equation 3 was originally derived by considering high-Reynolds number drag acting on a flat plate. As a more appropriate model for daphnids, we modified the derivation of Huntley and Zhou by considering the drag coefficient of a spherical particle, which yields an expression for total dissipated power 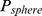 of:
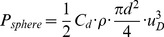
(4)


The drag coefficient 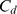 of a sphere for the range of 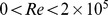 is given by:
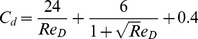
(5)


The above expression is equivalent to Stokes law if 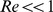 [35].

## Results

### Swimming kinematics and trail structure

The measured average size of the organisms were 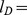 2.0 mm, 3.2 mm, and 3.5 mm in the ascending order of age groups. For each age group, four measurements of each swimming pattern were analyzed, hence, 36 trajectories were analyzed in detail (Table 1). The mean swimming velocity of the daphnids increased with increasing organism size from 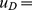 15 to 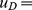 24 mm/s, resulting in 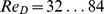.

**Table 1:** Summary of analyzed data and results.

**Age (days)**	5	20	35
**Size (** 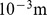 **)**	2.0 ( 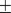 0.056)	3.2 ( 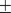 0.11)	3.5 ( 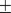 0.18)
**Speed (** 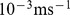 **)**	15.4 ( 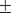 3.5)	18.2 ( 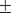 4.8)	23.6 ( 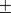 4.4)
**Reynolds number**	32 ( 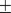 7.1)	58 ( 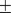 15.2)	84 ( 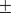 15.8)
**No. of observations (wakes, jets)**	12 (4, 8)	12 (7, 5)	12 (7, 5)
**Cruising (wakes, jets)**	(0, 4)	(3, 1)	(2, 2)
**Hopping and sinking (wakes, jets)**	(1, 3)	(1, 3)	(1, 3)
**Looping (wakes, jets)**	(3, 1)	(3, 1)	(4, 0)
**Mean trail volume (** 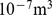 **)**	5.3 ( 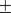 4.4)	10 ( 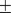 16.6)	60 ( 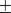 31)
**Mean trail length (** 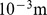 **)**	8.6 ( 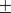 6.7)	14.1 ( 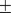 16.2)	56.3 ( 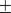 25.6)
**Mean trail diameter (** 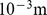 **)**	10.0 ( 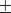 4.4)	9.5 ( 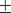 1.3)	12.9 ( 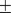 7.6)
**Mean dissipation rate (** 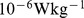 **)**	3.4 ( 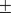 3.5)	2.4 ( 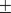 1.9)	1.8 ( 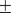 1.2)
**Mean total dissipated power (** 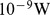 **)**	1.3 ( 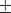 1.0)	1.3 ( 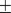 0.9)	10 ( 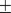 6.5)

Note that for the limited number of our observations, the chosen swimming patterns exhibited no systematic dependence with hydrodynamic quantities analyzed. Therefore, only the mean values of all quantified parameters irrespective of swimming pattern are presented with standard deviations within parentheses).

The observed flow fields in the hydrodynamic trails had two possible configurations differing in the directions of current velocity in the trail relative to swimming velocity of the daphnids. An actively swimming daphnid generates a propulsive jet, which is directed opposite to its swimming direction. Fluid drag acting on passively drifting *Daphnia*, on the other hand, generates a fluid wake, with flow velocities in the direction of daphnid motion. Both flow fields are exemplified in video S1 of the supplementary information. In both configurations, the trail can be described as a unidirectional and axisymmetric flow structure of cylindrical shape (see animated velocity distribution in the jet behind an upward swimming *Daphnia* in video S2 of the supplementary information). 50% of analyzed flow structures were wakes, and 50% jets (Table 1), irrespective of the pattern of swimming.

**Figure 5:**
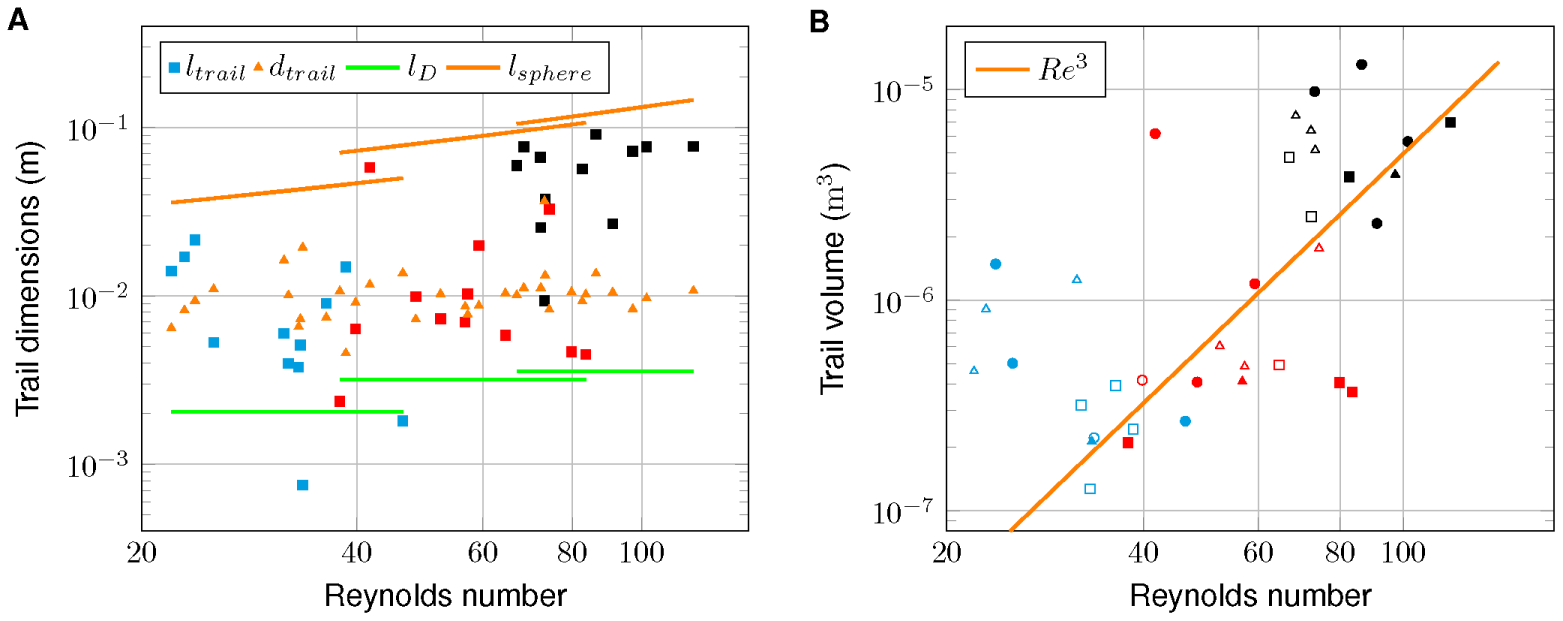
Observed trail dimensions vs. Reynolds number. (A) Observed trail length (squares) and trail diameter (triangles) vs. Reynolds number. Distinct colors of square markers indicate different age groups (cyan-5 days, red-20 days, and black-35 days). *Daphnia* length (green lines) and the length of corresponding sphere wakes (Equation 4) are shown for mean Reynolds number of the three age groups. (B) Observed trail volume vs. Reynolds number. Symbol color indicates age group, filled markers represent wakes and open markers jets. Distinct swimming patterns are indicated by square (cruising), triangle (hopping and sinking), and circle (looping). The line represents a proportionality to the 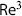.

The mean trail diameter in our observations was 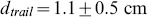 and did not vary with 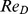 (Table 1, Figure 5A). The observed trail lengths varied over more than one order of magnitude also within the three size groups. For the largest animals considered in the study (i.e., 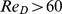), trail length exceeds the trail diameter approximately by a factor of ten. As shown in Figure 5, in particular the long trails of the largest organisms reach the lengths predicted by similarity scaling of laminar trails behind a translating sphere (Equation 6, Figure 5A). Increasing trail lengths lead to strongly increasing trail volume with increasing Reynolds number (Figure 5B). Observed trail volumes (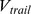) vary over two orders of magnitude and reaches up to 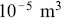, corresponding to about 500 times the body volume of the largest daphnids. While trail volume increases with the third power of Reynolds number (Figure 5B), no systematic dependence of trail dimension on flow configuration in the trail (wake or jet) or swimming pattern could be observed (Figure 5A).

### Trail energetics

**Figure 6:**
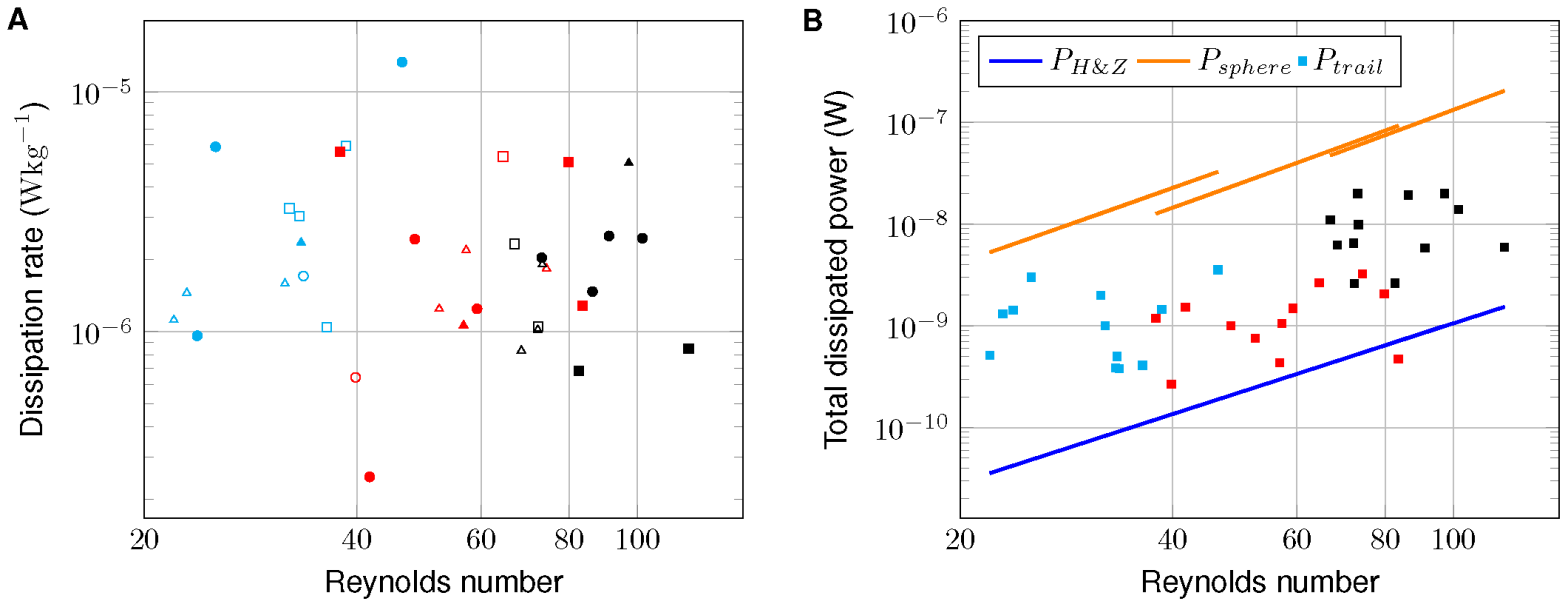
Trail-averaged viscous dissipation rates and total dissipated power within the trail vs. Reynolds number. (A) Trail-averaged viscous dissipation rates (total dissipated power/trail volume) vs. Reynolds number. Distinct colors indicate age groups (cyan-5 days, red-20 days, and black-35 days), filled markers represent wakes and open markers jets. Different swimming patterns are indicated by square (cruising), triangle (hopping and sinking), and circle (looping). (B) Total dissipated power within the trail vs. Reynolds number. Colors indicate age groups. The blue line shows dissipated power estimated according to Huntley and Zhou [9] (Equation 3), and the orange line the modified approach for a sphere (Equation 4).

Trail-averaged viscous dissipation rates varied between 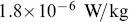 and 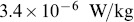 (Table 1). Similar to trail diameter, dissipation rates show no systematic dependence on Reynolds number, flow configuration, or swimming pattern (Figure 6A). As a result of increasing trail volume, total dissipated power within the trail, however, is increasing with Reynolds number (Figure 6B). Observed magnitudes of total dissipated power within the trail of the swimming daphnids varied between 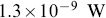 and 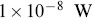. For our observations, the total dissipated power estimated according to Huntley and Zhou (Equation 3) provides a lower bound while the same approach applied to spherical organisms moving at intermediate Reynolds numbers (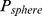, Equation 4) provides an upper bound (Figure 6B).

To assess the consistency of observed trail dimension and dissipated power, we estimated the length of an axis-symmetric trail of 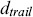 and of mean longitudinal velocity 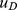, which is subject to viscous friction. Under these conditions, the power dissipated in the trail 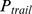 is equal to the product of viscous force acting on the cylindrical surface area of the trail and mean current velocity within the trail (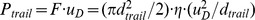). 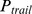 can further be expressed as the mean dissipation rate in the trail 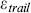 times trail volume (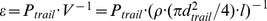), resulting in a relationship between trail length 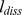 and mean kinetic energy dissipation within the trail:
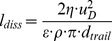
(6)


**Figure 7:**
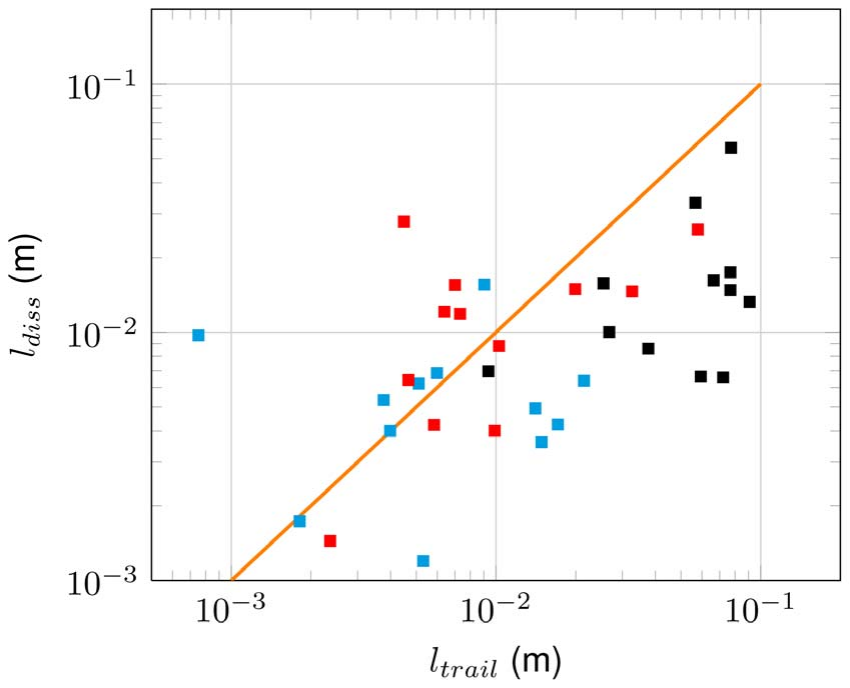
Trail length 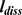 estimated from observed dissipation rates (equation 6) vs. observed wake length 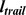. Distinct colors of square markers stand for age groups (cyan-5 days, red-20 days, and black-35 days) and the line represents a 1∶1 relationship.

Trail lengths estimated from Equation 6 using measured dissipation rates and trail diameter 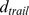 indicate a similar order of magnitude with observed trail lengths and also reproduce its dependence on *Daphnia* Reynolds number (Figure 7).

## Discussion

### Swimming speed, jets and wakes

**Figure 8:**
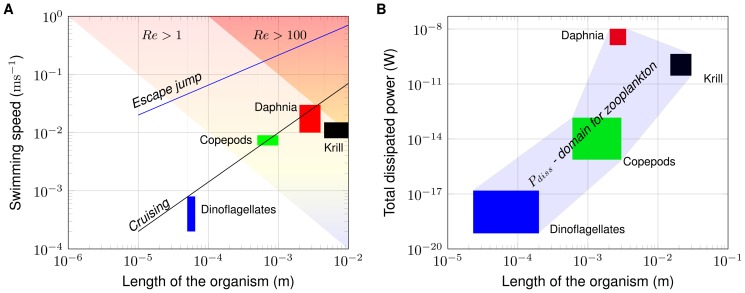
Comparison of *Daphnia* with other zooplankton. (A) Comparison of swimming speed. Regions for 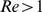 and 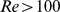 are denoted by light and dark pink colors. Lines show the empirical relationships for cruising and escape speeds for aquatic organisms as a function of organism size obtained by Huntely and Zhou [9]. Labeled boxes in color indicate the range of organism size and swimming speed for three zooplankton species considered in the analysis of Huntley and Zhou, as well as the present results for *Daphnia* (figure adopted from [10] with modifications). (B) Comparison of total dissipated power of *Daphnia* with empirical estimates for other zooplankton [9]. Labeled colored boxes indicate the range of organism size and dissipated power. Shaded area indicates a potential domain for dissipated power (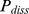) of kinetic energy produced by swimming zooplankton of different size.

Huntley and Zhou [9] have analyzed the hydrodynamics of swimming by 100 marine species ranging in size from bacteria to whales and found close empirical relationships between organism Reynolds number and cruising (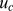), and escape (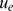) swimming speeds, respectively. These relationships have been converted to functions of body size by Kunze [10], resulting in 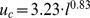 and 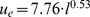. While having body length in between those of copepods and krill, the observed swimming velocities of *Daphnia* closely follow the relationship for cruising speed, (Figure 8A). The close correspondence to 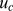 is expected, because escape behavior was not observed in our measurements. Nevertheless, daphnids are capable of performing escape reactions by increasing antenna beat frequencies up to five-fold (up to 23 Hz [41]).

The hydrodynamic footprint of *Daphnia* can either be a propulsive jet, which is generated by antenna motion and entrainment of ambient fluid [20], [41], or it can be a wake of fluid dragged behind the translating body [42]. Although the direction of the current velocity relative to the direction of body translation (swimming direction) is reverse, we use the term trail for both flow configurations. Irrespective of the pattern of swimming, each age group exhibited an almost equal distribution of wakes and jets in our observations.

The direction of the trail does not yield a distinct pattern in the distributions of trail volume, dissipation rate, or total dissipated power. These findings indicate similar duration of time periods of active propulsion and of inertial or gravitational movement, as well as a comparable momentum balance between the traversing organism and their trail under both conditions. The direction of flow in the hydrodynamic footprints of *Daphnia* therefore does not provide directional information for locating the organism and potentially reduces the risk of predation. However, the exact information about the nature of a predator's perception of hydrodynamic cues is not known, therefore, the intensity of the signal may still provide guidance to the source.

Our video S2 shows that a body vortex is formed around the body of the swimming *Daphnia*. Though the analyses of these vortices are beyond the scope of our work as we focused on the far-field, formation of similar vortex rings have been observed for other organisms and spheres [43]–[45]. In the case of repositioning jump made by copepods, two counter-rotating viscious vortex rings are formed in the wake and around the body of the copepod [43]. Shedding of conspicuous vortices during escape jumps of copepods have also been reported elsewhere, copepod mechanoreceptors can detect jet-like wakes produced by their preys during escape hops in the vortex flow field initially created by copepods [44]. In a separate study, the flow vorticity produced in a feeding current by copepods increases along the antennae [45].

### Trail size and energy dissipation

Our results for the trail dimensions are in good agreement with the empirical estimations suggested by [20]. Using their empirical formulae provided for the trail volume at the lowest density gradient (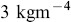) that they have used and considering a fully developed trail (i.e. after 6 s of the trail evolution) of *Daphnia* having a length of 2.12 mm, the trail volume is approximately 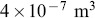. This is closely comparable to the mean trail volume estimated in our study (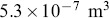) for *Daphnia* with 2 mm length (Table 1). Similarly, the trail length estimated using the empirical formulae by [20] for the same density gradient is about 8.3 mm whereas our estimations yielded a mean trail length of 8.6 mm (Table 1) for similar *Daphnia* size. However, the estimations using the approach by [20] should still be corrected to avoid underestimating the trail dimensions, because their lowest density gradient is still large. Assuming that the maximum length of the trail remains constant at gradients less than 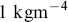 [20], trail length is about 10.6 mm at smaller gradients which overestimates our estimations of the mean trail length by a factor of 1.2. The discrepancy can be attributed to the different species ([20] used *Daphnia pulicaria*), larger fluctuations of trail length at gradients less than 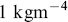, and other unknowns.

With increasing Reynolds number, an increasing fraction of power is dissipated at scales significantly exceeding the size of the organism. For the observed range of Reynolds number, our results indicate that the length of the trail increases whereas the width of the trail remains approximately constant. This is in accordance with velocity scaling in a laminar sphere wake [40], which predicts that velocity perturbations decrease exponentially in the radial direction and reciprocally along the centerline of a wake. Increasing trail length, due to increasing inertia of the displaced fluid at higher Reynolds number, results in an increase of the fraction of power dissipated in the trail. The category of swimming pattern or the trajectory of swimming neither affects the size nor the energetics of the trails.

Our measurements did show similar dissipation rates of kinetic energy for the different patterns of swimming. While trail sizes and energetics being independent of swimming patterns may be ascribed to the unique propulsion mechanism of *Daphnia* (beating their antennas), it still poses the question why organisms choose different swimming patterns. In addition to dissipate energy, the organisms need to perform other functions that may demand to differ their trajectories. In order to seize a patch at a faraway distance, organisms may need to cruise to travel a greater distance within a short time [46]. Moreover, cruising may also help to evade slower moving predators. Looping may be required to search for a good food patch in the neighborhood of the organism and remain in the patch during its area-restricted food search [47]–[49]. Looping can be additionally useful to escape from predators that largely rely on linear perception. Hop and silent sinking may need to perform evasive actions to evade mechanoreceptive predators.

Total dissipated power measured in the trail of swimming daphnids exceeds the estimates of Huntley and Zhou [9] by one to two orders of magnitude (Figure 8B). Also, the total dissipated power estimated by Huntley and Zhou [9] for different zooplankton species along with the total dissipated power measured in the trail of swimming daphnids provide a potential likelihood region of dissipated power for zooplankton (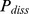 in Figure 8B), and the total dissipated power measured in the trail of swimming daphnids exceeds the smallest zooplankton species considered by about 10 orders of magnitude. Applying the theoretical approach of Huntley and Zhou to a moving sphere, instead of a flat plate, and using a drag coefficient which takes low-Reynolds number effects into account (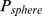, Equation 4), results in much higher estimates of power (Figure 6B). It should be noted, that our measurements, which result in power estimates in between those of Huntley and Zhou [9] and 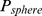, did not resolve the entire power dissipated in the flow field due to limitations in resolving velocity gradients in close proximity of the organism. Particularly at low Reynolds numbers, most of the energy is dissipated in close proximity of the organism. Hence, our measurements potentially underestimate total dissipated power and energy expenditure of swimming daphnids. However, they provide estimates of power, which is dissipated at larger spatial scales, exceeding the size of the animal by more than a factor of ten.

Mean volume, dissipation rate and total dissipated power of the 5 and 20 days old daphnids in the current study correspond to the values Noss and Lorke [24] observed in the trail of an approximately 4 mm large *Daphnia magna* swimming in a weak density stratification of 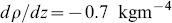. Although [20] observed that a weak stratification (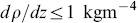) has negligible effects on the trail length of swimming *Daphnia*, the density stratification in Noss and Lorke [24] might be the main reason for the difference in comparison to our values obtained for the 35 days old daphinds. Numerical simulations of Ardekani and Stocker [50] revealed that the flow field and fluid transport generated by similar sized organisms is affected also by weak density stratification.

### Implications for biomixing

The significance of zooplankton-induced fluid motion for vertical mixing of density-stratified waters at larger scale has been the subject of a recent scientific debate. While different modeling approaches suggest significant contributions to energy production [8], [9] and also fluid mixing [51], [52], particularly the latter has been questioned based on scaling arguments [10], [11]. These arguments were based on the assumption that the spatial extent of the hydrodynamic disturbances produced by zooplankton is comparable to organism size. This assumption is only valid for low Reynolds number flow (i.e. 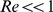). The turbulent drag law applied by Huntley and Zhou [9], in contrary, is only valid for high Reynolds number flow (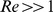). Swimming of most zooplankton organisms, however, is associated with Reynolds numbers in the transitional range [53]–[55], where both approaches are not valid. Our measurements show that in the hydrodynamic trails of *Daphnia*, kinetic energy is dissipated at rates even exceeding current estimates. This finding indicates that more detailed numerical simulations of fluid transport by swimming organisms obtained for Stokes flow [7], [56], [57] (neglecting fluid inertia and trails) cannot unrestrictedly be applied to *Daphnia* and potentially other zooplankton species swimming at intermediate Reynolds number.

Enhanced dissipation rates were observed at spatial scales about 10-fold larger than the size of the organisms. Kinetic energy dissipation is associated with current shear, which also enhances small-scale gradients of dissolved substances. The corresponding mixing, i.e. the dissipation rate of concentration variance by molecular diffusion, can be described by an apparent (eddy) diffusivity. The quadratic dependence of eddy diffusivity on the size of fluid disturbances [10] results in a 100-fold increase of diffusivity and scalar mixing rates if the size of the hydrodynamic trail instead of the commonly [10], [11] applied organism size is considered. Because molecular diffusivities of dissolved substances are about three orders of magnitude smaller than the diffusivity of momentum, concentration variance is dissipated more slowly than velocity variance. This results in larger dimensions of the trail behind the swimming organisms if it is defined based on concentration measurements. Using a fluorescent tracer, Noss and Lorke [15] have estimated the size of the trail behind swimming daphnids in terms of dissipation rates of concentration variance to be between 1 and 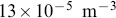, about one hundred times larger than the hydrodynamic trails observed in the present study. The relevant size of the hydrodynamic trail is defined by the particular context and can be expected to differ strongly, e.g., for hydromechanical and chemical signaling.

## Supporting Information

Figure S1 **An example of a trail produced by a cruising** ***Daphnia*** **.** The *Daphnia* swims in the negative z-direction, and blue dots indicate locations where dissipation rates exceed the selected threshold. The method illustrated in Figure 4 was used in the computation of the trail.
(TIF)

Table S1 **Impact of green laser light on organism swimming behavior.** Incoming and outgoing angles of the trajectories with respect to the light sheet were estimated for 4 observations per each swimming pattern and age group (i. e., 36 observations in total). The standard deviations of angular differences are shown within parentheses. We found that the difference between these angles remains similar for cruising while the differences of angles for hopping & sinking and looping are within an acceptable range. It should be noted that hopping & sinking and looping are naturally inclined to change the swimming direction. The relatively higher angle for hopping & sinking of 5 days old organisms can be due to switching between hopping and sinking within the width of the light sheet. This implies that the green laser light does not have any major implications that may have lead the organisms to veer from their original pathways. Nevertheless, the presence of the green laser light may affect the organism outside the vicinity of the laser light sheet.
(PDF)

Video S1 **A sample video illustrating the currents induced by** ***Daphnia*** **(video file format: avi).** Fluid motion is solely caused by swimming *Daphnia*. Frame dimensions are **32** by **32** mm. The video, which was recorded by one of the PIV cameras during our preliminary measurements, shows daphnids of various sizes crossing the laser light sheet. The flow field can be observed visually by following the white seeding particles used for PIV measurements. Judging by the dispersion of seeded particles at each crossing of *Daphnias*, it can be convincingly noted that a sudden leap of induced currents is associated with larger *Daphnias*. The video additionally illustrates that even the passive sinking of larger *Daphnias* can induce much larger trails than those of smaller *Daphnias* swimming at a high speed.
(AVI)

Video S2 **Animated three-dimensional vector plot of current velocities illustrating the propulsive jet induced by an upward swimming** ***Daphnia*** **(yellow blob) (video file format: avi).** The video is based on a single measurement using tomographic PIV conducted at LaVision. The tomographic reconstruction of the volumetric intensity distribution was performed using an implementation of the MART algorithm [58] and the velocity field was calculated using 3D correlation (*DaVis 8* by LaVision).
(AVI)
