# Supplementary figures and images for: Hydrodynamic Trails Produced by Daphnia: Size and Energetics
Source: PLoS One. 2014 Mar 26;9(3):e92383. doi: 10.1371/journal.pone.0092383 (PMC3966788; doi:10.1371/journal.pone.0092383)

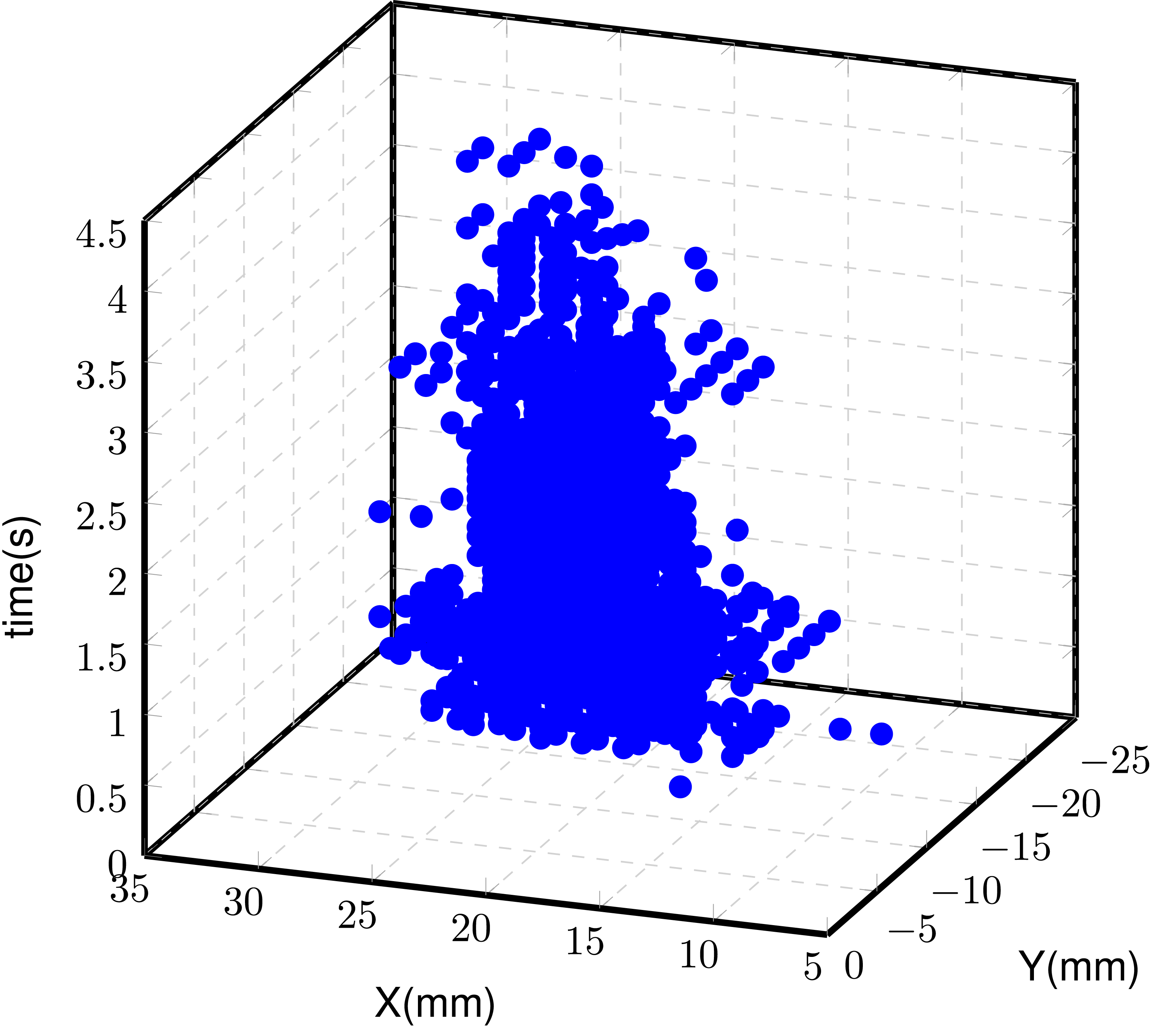

Supplement: Figure S1 — An example of a trail produced by a cruising Daphnia . The Daphnia swims in the negative z-direction, and blue dots indicate locations where dissipation rates exceed the selected threshold. The method illustrated in Figure 4 was used in the computation of the trail. (TIF) [file pone.0092383.s001.tif]
