# Supplementary material for: Hydrodynamic Trails Produced by Daphnia: Size and Energetics
Source: PLoS One. 2014 Mar 26;9(3):e92383. doi: 10.1371/journal.pone.0092383 (PMC3966788; doi:10.1371/journal.pone.0092383)
Supplement: Table S1 — Impact of green laser light on organism swimming behavior. Incoming and outgoing angles of the trajectories with respect to the light sheet were estimated for 4 observations per each swimming pattern and age group (i. e., 36 observations in total). The standard deviations of angular differences are shown within parentheses. We found that the difference between these angles remains similar for cruising while the differences of angles for hopping & sinking and looping are within an acceptable range. It should be noted that hopping & sinking and looping are naturally inclined to change the swimming direction. The relatively higher angle for hopping & sinking of 5 days old organisms can be due to switching between hopping and sinking within the width of the light sheet. This implies that the green laser light does not have any major implications that may have lead the organisms to veer from their original pathways. Nevertheless, the presence of the green laser light may affect the organism outside the vicinity of the laser light sheet. (PDF) [file pone.0092383.s002.pdf]

| Age (days) | Incoming-outgoing angle difference (°) | | |
|---|---|---|---|
| | **Cruising** | **Hopping & sinking** | **Looping** |
| 5 | 3.02 (±3.0) | 22.44 (±15.4) | 8.34 (±5.9) |
| 20 | 7.45 (±1.7) | 3.36 (±2.2) | 13.14 (±6.8) |
| 35 | 1.90 (±1.3) | 8.18 (±3.0) | 11.32 (±6.8) |
